# Development of a rapid knee cartilage damage quantification method using magnetic resonance images

**DOI:** 10.1186/1471-2474-15-264

**Published:** 2014-08-06

**Authors:** Ming Zhang, Jeffrey B Driban, Lori Lyn Price, Daniel Harper, Grace H Lo, Eric Miller, Robert J Ward, Timothy E McAlindon

**Affiliations:** 1 Division of Rheumatology, Tufts Medical Center, 800 Washington Street, Box #406, Boston, MA 02111, USA; 2 The Institute for Clinical Research and Health Policy Studies, Tufts Medical Center, and Tufts Clinical and Translational Science Institute, Tufts University, 800 Washington Street, Box #63, Boston, MA 02111, USA; 3 Medical Care Line and Research Care Line; Houston Health Services Research and Development (HSR&D), Center of Excellence Michael E. DeBakey VAMC, Houston, TX, USA; 4 Section of Immunology, Allergy, and Rheumatology, Baylor College of Medicine, Houston, TX. 1 Baylor Plaza, BCM-285, Houston, TX 77030, USA; 5 Department of Electrical and Computer Engineering, Tufts University, 216 Halligan Hall, Medford, MA 02155, USA; 6 Department of Radiology, Tufts Medical Center, 800 Washington Street, Box #299, Boston, MA 02111, USA

**Keywords:** Osteoarthritis, Cartilage, Quantitative measurements, Informative locations, Magnetic resonance imaging

## Abstract

**Background:**

Cartilage morphometry based on magnetic resonance images (MRIs) is an emerging outcome measure for clinical trials among patients with knee osteoarthritis (KOA). However, current methods for cartilage morphometry take many hours per knee and require extensive training on the use of the associated software. In this study we tested the feasibility, reliability, and construct validity of a novel osteoarthritis cartilage damage quantification method (Cartilage Damage Index [CDI]) that utilizes informative locations on knee MRIs.

**Methods:**

We selected 102 knee MRIs from the Osteoarthritis Initiative that represented a range of KOA structural severity (Kellgren Lawrence [KL] Grade 0 – 4). We tested the intra- and inter-tester reliability of the CDI and compared the CDI scores against different measures of severity (radiographic joint space narrowing [JSN] grade, KL score, joint space width [JSW]) and static knee alignment, both cross-sectionally and longitudinally.

**Results:**

Determination of the CDI took on average14.4 minutes (s.d. 2.1) per knee pair (baseline and follow-up of one knee). Repeatability was good (intra- and inter-tester reliability: intraclass correlation coefficient >0.86). The mean CDI scores related to all four measures of osteoarthritis severity (JSN grade, KL score, JSW, and knee alignment; all p values < 0.05). Baseline JSN grade and knee alignment also predicted subsequent 24-month longitudinal change in the CDI (p trends <0.05). During 24 months, knees with worsening in JSN or KL grade (i.e. progressors) had greater change in CDI score.

**Conclusions:**

The CDI is a novel knee cartilage quantification method that is rapid, reliable, and has construct validity for assessment of medial tibiofemoral osteoarthritis structural severity and its progression. It has the potential to addresses the barriers inherent to studies requiring assessment of cartilage damage on large numbers of knees, and as a biomarker for knee osteoarthritis progression.

## Background

Measurement of hyaline cartilage damage is viewed as a primary endpoint in the assessment of structural progression of knee osteoarthritis (OA). However, the traditional radiographic measurement approach provides only indirect visualization of cartilage and is limited by poor reproducibility and sensitivity to change [1]. Magnetic resonance (MR) imaging is a noninvasive technology that can generate 3-dimensional images of intra-articular soft-tissue structures, including hyaline cartilage. Quantification of knee cartilage morphology (e.g., thickness, volume) is highly reliable and provides potential surrogate endpoints for epidemiologic studies and clinical trials of interventions with potential for structure modification [2-6]. However, the process of measuring cartilage morphology on MR images is time-consuming and burdensome. Each 3-dimensional (3D) knee MR sequence may take many hours for a reader to manually segment. Furthermore, operators who use cartilage segmentation software often need extensive training [7] which further contributes to the time and cost.

Over the past decade, researchers have deployed several approaches to reduce the burden of measuring knee cartilage on MR images. These have included segmenting alternate MR slices or confining measurements to partial regions of cartilage [8-10]. Computer-aided algorithms (e.g., active contours, B-splines) have also been developed to assist with cartilage segmentation [11-15]. Unfortunately, these methods lack sufficient accuracy and reliability to detect small cartilage changes [2]. Thus, there remains a need among researchers for a quantification method that can be rapidly computed and has good reproducibility, validity, and sensitivity to change.

The work in this paper is motivated by the observation that some articular cartilage locations are more susceptible to occurrence of OA damage and thus may be more informative in the measurement of its progression [16,17]. Thus, focusing effort on measuring these locations in a reproducible manner may improve the efficiency and sensitivity to change. Therefore, the goal of this study was to develop an efficient cartilage quantification method leveraging informative locations in the medial tibia and femur, and to test its reliability, construct validity, and sensitivity to change. We focused on the medial tibiofemoral compartment because medial OA is more common than lateral OA [18,19].

## Methods

### Rapid knee cartilage damage index quantification method

**Figure 1:**
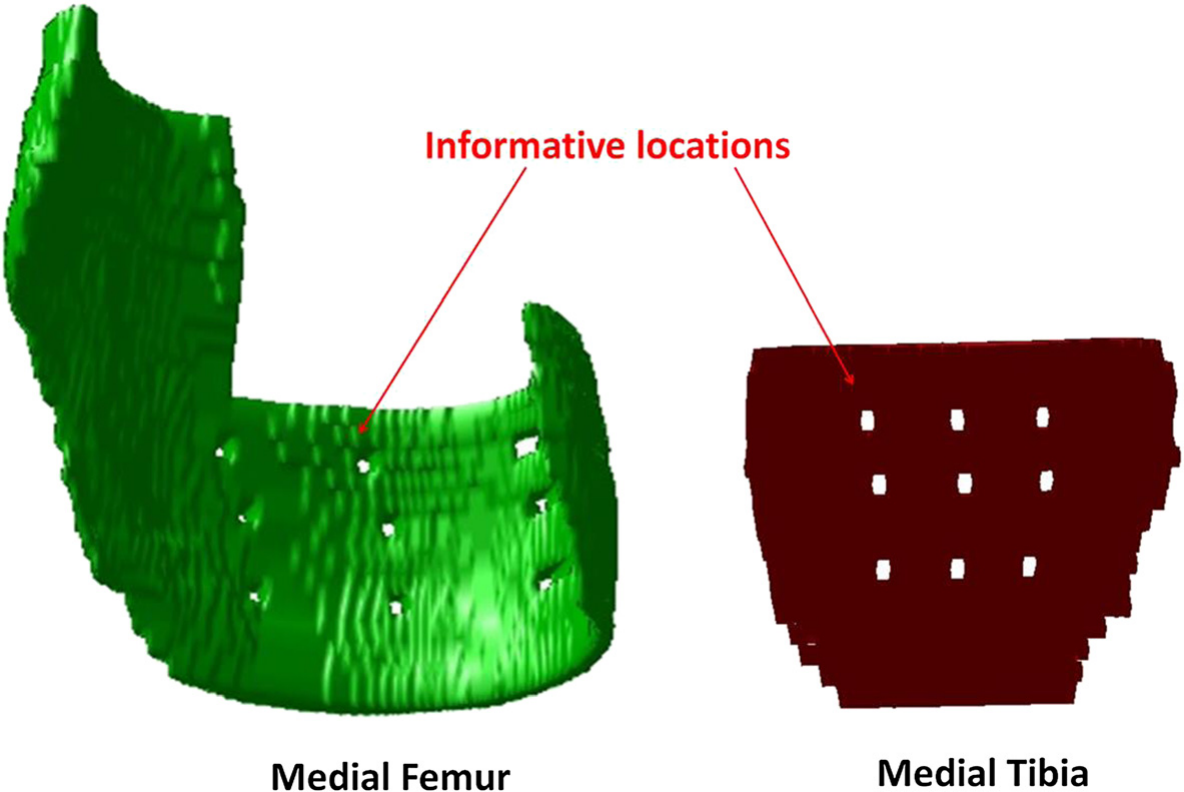
3D medial cartilage images with 9 informative locations on femur and 9 informative locations on tibia (the 3D images were rotated for better viewing informative locations).

We developed a rapid knee cartilage damage quantification method using knee MR images from three datasets (263 knees). Underlying this methodology is a 2-dimensional, rectangular, universal coordinate systems to represent the articular surface of the distal femur and proximal tibia. Using previously manually segmented knees [20], we projected the denuded cartilage area on our coordinate system to identify the areas in the joint surface that are most frequently denuded of cartilage. Based on the results of that analysis, we then selected nine locations within the region of the most commonly denuded areas on the medial femur and tibia (Figure 1). A full description of the developmental methodology and related data is provided in the Additional file 1.

### Validation dataset

For this validation study we used data and MR images from the Osteoarthritis Initiative (OAI), which was initiated to promote the evaluation of OA biomarkers as potential surrogate endpoints [4]. The OAI has institutional review board approval (IRB) from the coordinating centers and the four clinical centers (University of Maryland and John’s Hopkins comprise a single recruitment center, Brown University, Ohio State University, University of Pittsburgh). All participants provided informed consent to participate in the OAI. The four OAI clinical centers recruited approximately 4800 men and women (ages 45–79 years) with or at risk for knee OA. The OAI participants had weight-bearing posterior-anterior fixed-flexion knee radiographs obtained at the baseline and 24-month visits. We obtained a convenience sample of 102 pairs of knee (baseline and 24-month MR scans) not included in our developmental datasets that had complete data (i.e., clinical, static knee alignment, semi-quantitative radiographic grading, and joint space width). We selected our sample to represent the range of radiographic OA severity (Kellgren-Lawrence [KL] scores 0 to 4) enriched with knees that showed radiographic worsening over time. We randomly selected 40 knees with KL = 2, among whom 20 knees had worsening of their KL grade over 24 months, and 20 knees that did not. We also randomly included 35 knees with KL = 3, among whom 15 knees increased KL grade over 24 months and 20 knees did not change. We included all of the knees with KL grades 0, 1, and 4 that had complete data and were not included in our developmental datasets.

### MR image assessments

Our validation analyses used the OAI 3D sagittal water-excitation dual-echo steady state (DESS) images with field of view = 140 mm, slice thickness = 0.7 mm, skip = 0 mm, flip angle = 25 degrees, echo time = 4.7 ms, recovery time = 16.3 ms, 307 × 384 matrix, phase encode ant/post. X resolution = 0.365 mm, and y resolution = 0.456 mm. All OAI images were obtained using one of four identical Siemens Trio 3-Tesla MR systems and a USA Instruments quadrature transmit-receive knee coil at one of four OAI clinical sites [21]. The OAI MR images are publicly available upon request at http://oai.epi-ucsf.org.

### Measurement of cartilage damage on MR images

**Figure 2:**
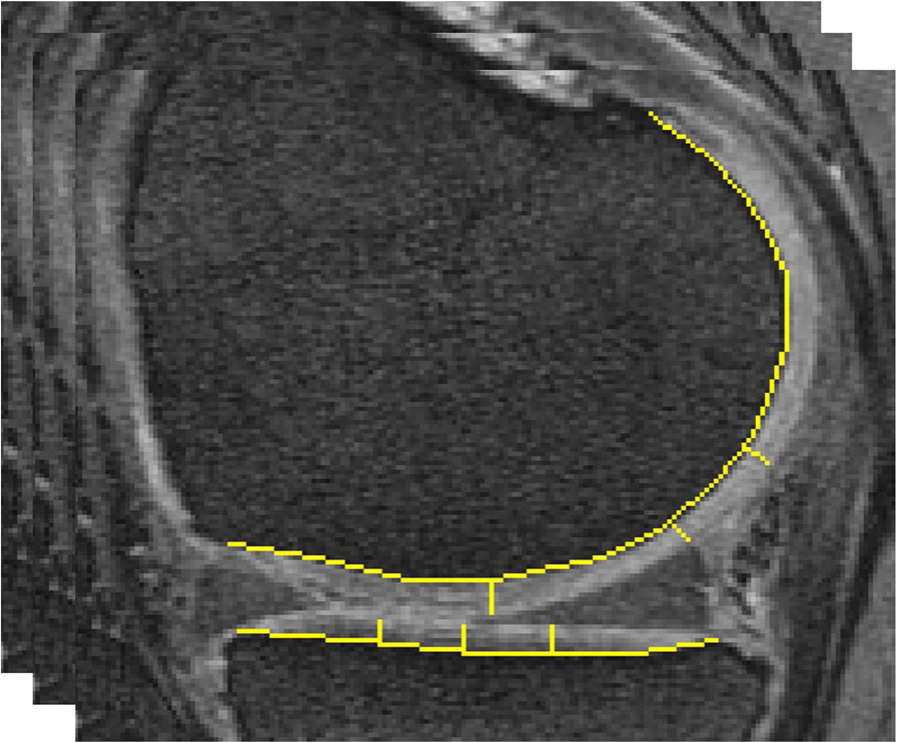
CDI measurement of the medial tibiofemoral compartment.

One investigator (MZ), who was blinded to the outcome measures, performed the CDI measurement on paired baseline and 24-month follow-up MR images. The investigator was not blinded to the order of images (baseline or follow-up). The investigator used customized software to (1) translate an articular surface into a 2-dimensional coordinate matrix, (2) localize 9 pre-defined informative locations (characterized by a greater propensity to exhibit cartilage loss), and (3) measure cartilage thickness at those locations (Figure 1). To co-locate the corresponding informative locations on baseline and follow-up images, we used dual screens to permit simultaneous visual comparison of the MR image sets.In the first step the reader indicated the most medial and lateral MR image slices within the knee. These images designated the minimum and maximum values of the medial-to-lateral axis on the 2-dimensional coordinate system. Next, the software automatically determined the MR image slices that contained the informative locations. On each of these slices the investigator manually traced the bone-cartilage boundary using predefined segmentation rules. The software then translated the length of the bone-cartilage boundary to a standardized anterior-to-posterior axis and indicated the predefined informative locations so that the investigator could measure the cartilage thickness at those points (Figure 2). The software then computed the cartilage damage index (CDI) by summing the products of cartilage thickness, cartilage length (anterior-posterior), and voxel size from each informative location.

### Assessment of reliability

To evaluate the intra- and inter-tester reliability we selected 20 pairs of knees (baseline and follow-up MR scans) representative of a full range of disease severity in the sample. Two investigators (MZ and DH) independently measured the CDI on two occasions, separated by at least 72 hours. We evaluated intra-tester and inter-tester reliability with intraclass correlation coefficients (ICC) [22]. Specifically, we used an ICC _3,1_ model for intra-tester reliability and an ICC _2,1_ model for inter-tester reliability. To explore if the reliability was consistent across levels of OA severity we conducted secondary analyses to explore ICCs among knees with no-mild medial joint space narrowing (JSN; medial JSN score = 0 or 1; n = 13) and more severe medial JSN (JSN score = 2 or 3; n = 7). We selected this JSN cut-point because it provided a sufficient sample size in each group to estimate ICCs.

### Assessment of measurement time

We recorded the measurement time for 20 pairs of knees (baseline and follow-up MR scans) and calculated the mean and standard deviation time to measure the 20 knees. The investigator started the timer when he started to load the MR images and stopped the timer after saving the quantification data.

### Radiographic assessments

To assess construct validity of the CDI we compared it with three radiographic measures of knee OA (JSN, KL grade, and JSW), that have been extensively reported in the past to be associated with cartilage damage [23-27]. The semi-quantitative and quantitative knee radiographic measurements have been previously described in detail [6,28-30]. Briefly, semi-quantitative assessments of radiographic knee OA severity were performed using the weight-bearing posterior-anterior fixed-flexion knee radiographs from the baseline and 24-month OAI visit. The central readers determined semi-quantitative scores [6] for KL grade and modified OARSI-atlas based assessment of medial JSN scores (0 to 3), which defined definite progression within an OARSI grade [31]. KL progression was defined as any worsening of the KL score from 0 to 24 months. A different group performed central measurements of joint space width (JSW) at fixed-locations in the medial tibiofemoral compartment. We selected JSW at one fixed location (x = 0.250) because it is one of the most responsive locations for assessing JSW change [28]. These data and descriptions of the methods are publicly available on the OAI website (kxr_sq_bu_00 [version 0.5] and kxr_sq_bu_03 [version 3.5], kxr_qjsw_duryea_00 [version 0.5] and kxr_qjsw_duryea_03 [version 3.4]; http://oai.epi-ucsf.org/; ICC > 0.93).

### Static alignment

To further assess construct validity of the CDI we tested its association with static knee alignment, a well-established risk factor for medial cartilage damage [23,27,32,33]. One reader measured static alignment, hip-knee-ankle (HKA) angle on standing full-limb radiographs that were collected at either the 12- or 24-month OAI visit using a semi-automated program (developed by Jeff Duryea, ICC > 0.99).

### Analytic approach

We calculated descriptive characteristics for the sample. To account for different skeletal sizes, we adjusted the CDI by dividing the raw data by the participant’s height. Change in height-adjusted CDI was calculated as follow-up minus baseline. To explore the construct validity of the new cartilage quantification method we tested for a linear trend of baseline and change in CDI across higher baseline grades of JSN and KL. Linear trend was tested using linear regression and treating JSN and KL grades as continuous variables. We also used independent sample t-tests to determine if CDI change was different between knees with and without radiographic OA progression (based on changes in JSN and KL grades). Spearman correlations were used to assess the relationship between baseline and change in CDI to static alignment, baseline and 24-month change of JSW. P-values < 0.05 were considered statistically significant. To assess responsiveness among knees with and without radiographic OA progression we calculated absolute standardized response mean (SRM) values (SRM = mean change divided by standard deviation of change). All analyses were performed in SAS 9.3 (SAS Institute Inc., Cary, NC) with the exception of the ICCs, which were performed in SPSS 19 (IBM Co., NY).

## Results

### Validation dataset characteristics

The descriptive characters of 102 participants are in Table 1. There were 25 knees with medial JSN progression (different JSN grade at baseline and follow-up) and 39 knees with KL progression (different KL grade at baseline and follow-up). The overall SRMs for the CDI were −0.78 in the medial femur, −0.64 in the medial tibia, and −0.87 in the medial tibiofemoral compartment.

**Table 1:** Descriptive characteristics of validation dataset (n = 102)

**Age, mean (SD) years**	**61.61 (8.74)**	
**Female, n (%)**	56 (54.37)	
**JSN grade, n (%)**	**Baseline**	**24-month follow-up**
**0**	43 (42.16)	42 (41.18)
**1**	31 (30.39)	25 (24.51)
**2**	25 (24.51)	20 (19.61)
**3**	3 (2.94)	15 (14.71)
**KL score, n (%)**	**Baseline**	**24-month follow-up**
**0**	4 (3.92)	3 (2.94)
**1**	19 (18.63)	15 (14.71)
**2**	40 (39.22)	25 (24.51)
**3**	34 (33.33)	40 (39.22)
**4**	5 (4.90)	19 (18.63)

Note: n = number; SD = standard derivation; JSN = joint space narrowing; KL = Kellgren-Lawrence.

### Measurement time

The average CDI measurement time of 20 knees was 14.4 minutes (SD = 2.1) per pair of knees (baseline and 24 month scans).

### Assessment of reliability

Intra-tester (ICC_3,1_) reliability for both investigators ranged from 0.94 to 0.99. The good intra-tester reliability was consistent among knees with no-mild medial JSN (ICC_3,1_ = 0.93 to 0.99) and more severe JSN (ICC_3,1_ = 0.80 to 0.99). Inter-tester (ICC_2,1_) reliability for medial femur, medial tibia, and total medial tibiofemoral ranged from 0.86 to 0.95. The good inter-tester reliability was consistent among knees with no-mild medial JSN (ICC_2,1_ = 0.78 to 0.92) and more severe JSN (ICC_2,1_ = 0.87 to 0.93).

### Relationship of baseline CDI to baseline radiographic severity

#### Medial JSN

Knees with greater JSN score (i.e. greater OA severity) had lower mean medial femur, tibia, and tibiofemoral CDIs (indicating more cartilage damage; Table 2, all p for linear trend < 0.01).

**Table 2:** Cartilage damage index stratified by baseline medial joint space narrowing (JSN) grade

	**Baseline medial JSN Grade**				
**Cartilage measure**	**JSN = 0 (n = 43) mean (SD)**	**JSN = 1 (n = 31) mean (SD)**	**JSN = 2 (n = 25) mean (SD)**	**JSN = 3 (n = 3) mean (SD)**	**p-value for trend**
	**Cross-sectional**				
**Femur CDI (Baseline)**	825.14 (148.42)	754.71 (125.54)	529.29 (130.35)	325.89 (324.51)	<0.01
**Tibia CDI (Baseline)**	352.29 (72.47)	328.96 (60.91)	233.45 (68.77)	95.83 (99.73)	<0.01
**Tibiofemoral CDI (Baseline)**	1177.40 (198.78)	1083.70 (173.17)	762.74 (183.50)	421.72 (420.65)	<0.01
	**Longitudinal**				
**Femur CDI (Change)**	−31.06 (46.40)	−49.67 (61.65)	−78.55 (66.81)	−185.4 (170.07)	<0.01
**Tibia CDI (Change)**	−12.17 (25.16)	−23.27 (33.83)	−46.18 (52.46)	−42.41 (41.07)	<0.01
**Tibiofemoral CDI (Change)**	−43.23 (57.65)	−72.94 (76.02)	−124.70 (97.47)	−227.80 (208.59)	<0.01

Notes: change = follow-up minus baseline; SD = standard deviation.

#### JSW measurement

The baseline medial femur, tibia, and tibiofemoral CDI scores were significantly correlated with baseline JSW (Table 3; all p < 0.05).

**Table 3:** Correlation between CDI and baseline HKA and JSW

**Spearman correlation**		
	**HKA**	**JSW baseline**
**Femur CDI (Baseline)**	0.34*	0.84*
**Tibia CDI (Baseline)**	0.21*	0.77*
**Tibiofemoral CDI (Baseline)**	0.31*	0.87*
**HKA JSW change**		
**Femur CDI (Change)**	0.29*	0.30*
**Tibia CDI (Change)**	0.34*	0.40*
**Tibiofemoral CDI (Change)**	0.36*	0.43*

Notes: *p < 0.05; HKA = hip-knee-ankle; JSW = joint space width change = follow-up minus baseline.

#### KL grade

There was generally a lower CDI across increasing KL, with the exception that knees with KL grade 2 had a greater CDI compared to those with KL grade 1 (Table 4, all p for linear trend < 0.01).

**Table 4:** Cartilage damage index stratified by baseline Kellgren-Lawrence (KL) grade

**Cartilage measure**	**KL = 0 (n = 4) mean (SD)**	**KL = 1 (n = 19) mean (SD)**	**KL = 2 (n = 40) mean (SD)**	**KL = 3 (n = 34) mean (SD)**	**KL = 4 (n = 5) mean (SD)**	**p-value for trend**
	**Cross-sectional**					
**Femur CDI (Baseline)**	771.99 (157.40)	750.75 (159.50)	781.47 (114.73)	634.00 (218.90)	583.92 (434.01)	<0.01
**Tibia CDI (Baseline)**	363.07 (103.74)	314.96 (60.46)	343.96 (66.72)	272.83 (94.26)	199.81 (163.56)	<0.01
**Tibiofemoral CDI (Baseline)**	1135.1 (257.41)	1065.7 (207.06)	1125.4 (155.11)	906.83 (304.59)	783.73 (595.54)	<0.01
	**Longitudinal**					
**Femur CDI (Change)**	−81.06 (46.06)	−30.35 (43.25)	−36.72 (59.38)	−72.92 (62.13)	−109.4 (159.37)	0.02
**Tibia CDI (Change)**	−56.37 (43.48)	−17.85 (32.13)	−15.14 (26.74)	−35.36 (49.33)	−30.86 (33.75)	0.40
**Tibiofemoral CDI (Change)**	−137.40 (80.56)	−48.19 (61.81)	−51.86 (69.27)	−108.30 (90.93)	−140.20 (190.81)	0.03

Notes: change = follow-up minus baseline; SD = standard deviation.

#### Knee alignment

Baseline CDI was positively associated with baseline static knee alignment (Table 3). In other words, a lower CDI was associated with varus alignment.

### Relationship of longitudinal change in CDI to baseline radiographic severity

#### Medial JSN

Knees with greater baseline JSN score generally had greater subsequent change in the CDI (reflecting greater longitudinal cartilage loss; Table 2, all p for linear trend < 0.01). This trend plateaued at JSN = 2 for change in tibia CDI, but increased in a linear fashion for femur and tibiofemoral CDI change.

#### KL grade

There were less pronounced linear trends of change in the femur and tibiofemoral CDI across baseline KL scores. There was not a statistically significant trend found for change in tibia CDI (Table 4).

#### Knee alignment

There was a statistically significant relationship between longitudinal CDI over 24 months and baseline static alignment (Table 3); all p < 0.05, such that those with more varus alignment had more medial cartilage damage longitudinally.

### Relationship of longitudinal change in CDI to longitudinal change in radiographic severity

#### Medial JSN and KL grades

Knees with radiographic progression over the 24 month observation period, as indicated by an increase in JSN or KL grade, had greater change in CDI scores compared with knees with no progression (Table 5). The SRM values for the CDI among knees with JSN or KL change (i.e. those with structural progression) were 12% to 300% greater than knees without JSN or KL change (e.g., SRM = −1.39 for tibia JSN progression knees; SRM = −0.45 for tibia JSN non-progression knees).

**Table 5:** Change in CDI among knees with and without structural progression

	**Joint Space Narrowing (JSN)**								
	**No structural progression**				**Structural progression**				**p-value**
	**n**	**Mean**	**SD**	**SRM**	**n**	**Mean**	**SD**	**SRM**	
**Femur CDI (Change)**	74	−35.73	50.14	−0.71	25	−87.82	68.62	−1.28	<0.0001
**Tibia CDI (Change)**	74	−10.86	23.99	−0.45	25	−63.83	46.02	−1.39	<0.0001
**Tibiofemoral CDI (Change)**	74	−46.58	57.48	−0.81	25	−151.7	90.95	−1.67	<0.0001
	**Kellgren-Lawrence (KL)**								
	**No structural progression**				**Structural progression**				**p-value**
	**n**	**Mean**	**SD**	**SRM**	**n**	**Mean**	**SD**	**SRM**	
**Femur CDI (Change)**	58	−40.53	49.84	−0.81	39	−64.06	70.06	−0.91	0.060
**Tibia CDI (Change)**	58	−15.86	33.21	−0.48	39	−37.23	43.34	−0.86	0.007
**Tibiofemoral CDI (Change)**	58	−56.39	64.12	−0.88	39	−101.3	96.68	−1.05	0.007

Notes: Structural Progression, JSN/KL has different grade at baseline and follow- up; N = number of knees (the numbers were excluded JSN = 3 and KL = 4 since both of them are the highest grade, no grade change happens); SD = standard deviation; SRM = standardized response mean.

#### JSW measurement

The 24-month change of medial femur, tibia, and tibiofemoral CDI scores were significantly correlated with 24-month change of JSW (Table 3; all p < 0.05)

## Discussion

This study demonstrates that the MR-based CDI can be rapidly and reliably applied in the medial tibiofemoral compartment, and has construct validity as an aggregate measure of cartilage damage in knee OA. The predicate of the development of the CDI was that a focus on locations that have increased susceptibility to cartilage loss would shorten measurement time and increase sensitivity to change [16,34], a notion that our results appear to corroborate. As a method that can be rapidly deployed, it offers the potential to address the current barriers that measuring OA cartilage damage on large numbers of knee MR images. With apparently good discriminative validity for worsening of knee OA structural severity, it may also have usefulness as a proxy biomarker of OA progression.

We found that the CDI can be measured in the medial tibiofemoral compartment by a trained technician within about 14 minutes for a pair of knee images. While future development of the methodology will need to expand the CDI into the lateral tibiofemoral and patellofemoral compartments, the total measurement time will likely remain substantially shorter than for other MR-based cartilage measurement methods.

We tested the construct validity of the CDI by comparing it with other established radiographic measures of knee OA severity including medial tibiofemoral JSN (a semi-quantitative scale), JSW (continuous), KL grade (a global semi-quantitative score), and knee alignment. Radiographic JSN and JSW are generally attributed to loss of articular cartilage among knees with OA [35]. One study found that knees with JSN = 2 and JSN = 3 have 27% and 56% less cartilage thickness in the central medial tibiofemoral region compared with a contralateral knee with no JSN [23]. While we only used 18 informative locations, our CDI detected a similar trend with 35% and 64% less medial tibiofemoral CDI among knees with JSN = 2 and 3 (Table 2) compared with knees without JSN. Furthermore, prior studies have found similar correlations to ours for medial JSW and medial tibiofemoral cartilage morphology (r = 0.46 to 0.71) [27,36,37] and changes in these measures (r = 0.21 to 0.48) [24,25]. Overall, we consistently found relationships between the CDI and the severity of radiographic OA except that knees with KL grade 2 had a greater baseline CDI and less apparent progression compared with those with KL grade 1 (i.e., suggesting less damage; Table 4). However, others have observed that knees with KL grade 2 often have thicker cartilage and less cartilage loss, which is attributed it to cartilage swelling -- an early feature of cartilage damage [26,27]. Despite the CDI being based on only 18 locations, these informative locations are sufficient to calculate a CDI that agrees with pre-established associations between cartilage damage and radiographic OA severity.

In addition to verifying that the CDI was associated with radiographic OA severity we also demonstrated that the CDI is related to knee alignment (baseline CDI: r = 0.21 to 0.33, CDI change: r = 0.29 to 0.36), which is a strong risk factor for knee OA progression [27,32]. Our correlations are comparable to other MR-based cartilage measures (cross-sectional r = 0.20 to 0.22; change in cartilage r = 0.10 to 0.40) [23,33]. These findings further support that not only is the CDI associated with other measures of disease severity but also an important risk factor.

In a recent meta-analysis of articular cartilage quantitative assessments that provided SRMs to reflect responsiveness, measures of the medial tibia had SRMs that ranged from −0.63 to −0.34, medial femur: −0.74 to −0.28, and medial tibiofemoral: −1.26 to −0.46 [38]. The SRMs for the CDI (Table 5) were comparable to those reported in this recent systematic review. This was true even among knees without structural progression with the exception of the medial tibia. These findings suggest that the CDI performs well on cartilage measurements; however, additional studies are needed to directly compare these methods.

There are a number of limitations to our study including that that the 3D DESS sequence in the OAI utilized a low flip angle of 25°, which is not optimal for contrasting fluid and cartilage signal [39]. However, the OAI DESS sequence is well validated [4,40], and has been successfully used to measure cartilage volume many studies using traditional manual segmentation methods [4,23,26,41-44]. Therefore, the OAI was a reasonable data set in which to develop the CDI measurement and it is quite possible that the CDI may perform even better in other data sets. Another limitation to our study was that our validation data set did not include total cartilage segmentation values. The CDI, however, was developed using manual cartilage segmentation (see Additional file 1) where we found a good correlation (r > 0.60, Additional file 1: Table S3) between baseline CDI and cartilage volume (manual segmentation). It may be advantageous to test the association between CDI and manual segmentation in a different dataset to verify that the CDI is related to cartilage volume beyond the OAI. Another limitation was that we did not quantify the accuracy of placing the informative locations. We believe that the placement of informative locations on baseline and follow-up images was accurate because we used a robust coordinate system, trained the reader to detect errors, found good construct validity, and detected large measures of responsiveness. Future studies should quantify the accuracy of placing the informative locations and strive to minimize any error since this may further enhance the performance of the CDI. We would also point out that at present the CDI is only applicable to the medial tibiofemoral compartment. However, findings from this study reinforce the need to propagate the CDI approach to the lateral tibiofemoral and patellofemoral compartments. Finally, a potential drawback to the CDI is the possibility of failing to quantify cartilage damage at locations that were not identified as informative locations. This limitation is similar to other approaches that focus on specific regions of the articular surface (e.g., central weight-bear medial femur) [8-10]. Despite this limitation, the CDI performed well in these analyses, which may suggest that only a small number of knees, if any, experience considerable cartilage damage in regions not covered by the informative locations.

## Conclusions

In summary, this cartilage-damage quantification method, which is based on informative locations, is relatively easy to implement, provides reliable measurements, has good construct and discriminative validity, and is sensitive to change in the state of osteoarthritis. This method has the potential to address the current barriers that measuring OA cartilage damage on large numbers of knee MR images, such as the Osteoarthritis Initiative and other large epidemiologic investigations. Furthermore, with apparently good discriminative validity for worsening of knee OA structural severity, it may also have usefulness as a proxy biomarker of OA progression.

## Abbreviations

3D: 3-dimensional; CDI: Cartilage damage index; DESS: Dual-echo steady state; HKA: Hip-knee-ankle; ICC: Intraclass correlation coefficients; JSN: Joint space narrowing; JSW: Joint space width; KL: Kellgren Lawrence; MR: Magnetic resonance; MRIs: Magnetic resonance images; OA: Osteoarthritis; OAI: Osteoarthritis initiative; SD: Standard derivation; SRM: Standardized response mean.

## Competing interests

The authors have no competing interests that could potentially and inappropriately influence this work.

## Authors’ contributions

MZ participated in the conception and design of the study, acquisition of data, analysis, interpretation of data, drafting/revisions of the article, as well as final approval of the article. JBD participated in the conception and design of the study, acquisition of data, analysis and interpretation of data, drafting/revisions of the article, as well as final approval of the article. LLP participated in the conception and design, acquisition of data, analysis, interpretation of data, drafting/revisions of the article, as well as final approval of the article. DH participated in intra- and inter-tester reliability test. GHL participated in interpretation of data, drafting/revisions of the article, as well as final approval of the article.. EM participated in the conception and design, drafting/revisions of the article, as well as final approval of the article. RW participated in developing the cartilage segmentation rule, drafting/revisions of the article, as well as final approval of the article. TEM participated in the conception and design, analysis and interpretation of data, drafting/revisions of the article, as well as final approval of the article. All authors read and approved the final manuscript.

## Pre-publication history

The pre-publication history for this paper can be accessed here:

http://www.biomedcentral.com/1471-2474/15/264/prepub

## Supplementary Material

Additional file 1 Development of Cartilage Damage Index. Click here for file
