# Supplementary material for: Development of a rapid knee cartilage damage quantification method using magnetic resonance images
Source: BMC Musculoskelet Disord. 2014 Aug 6;15:264. doi: 10.1186/1471-2474-15-264 (PMC4126278; doi:10.1186/1471-2474-15-264)
Supplement: Additional file 1 — Development of Cartilage Damage Index. [file 1471-2474-15-264-S1.docx]

**Supplementary**

**Method**

We developed, tested, and validated the new cartilage damage quantification method in 4 steps using four separate datasets (See table 1). First, we performed primary development using manually segmented 1.5-Tesla MR images samples from a clinical trial of Vitamin D among patients with knee OA. In step 2, we transitioned the system to 3-Tesla magnetic resonance (MR) images derived from the Osteoarthritis Initiative (OAI) cohort. After primary development was completed, we refined the measurement decision rules (step 3). And finally (step 4), we used a unique set of MRIs to test the construct validity of cartilage damage index (CDI) score. Each step used unique datasets with the samples in step 2, 3, and 4 from the OAI. All of our studies are based on medial compartment of the knee.

*Step 1: Development*

*Dataset (n=83)*

The objective of the first phase was to design a coordinate system and determine the most informative locations for cartilage denudation. The development dataset was from a clinical trial among patients with symptomatic knee OA. The descriptive characteristics of the sample has been previously described (McAlindon JAMA 2013). The 3D DESS MR images were obtained on a Siemens Avanta 1.5-Tesla scanner using a transmit-receive extremity coil (slice thickness = 3mm, space thickness = 0.5mm, and field of view = 140mm). The cartilage segmentation procedures have been previously reported (Driban, BMC MSK, McAlindon JAMA 2013). Briefly, one reader manually delineated the 3D cartilage segments using ANALYZE (Biomedical Imaging Resource, Mayo Clinic; intra-reader reliability: read-re-read intraclass correlation coefficient [ICC] > 0.99). A second reader later marked areas of full thickness cartilage defects on each slice. The baseline and 24-month follow-up images were registered and specifically evaluated for cartilage loss.

*Procedure*

*Coordinate system*

We designed two 2-dimensional, rectangular, universal coordinate systems to represent the articular surface of the distal femur and proximal tibia. The coordinate systems were used to localize corresponding informative locations in baseline and follow-up knees. Within the coordinate system, the vertical axis represents the medial-to-lateral width of the articular surface (sagittal MR image slice sequence number) and the horizontal axis represents the anterior-to-posterior length of the articular surface.

Next, we projected the denuded cartilage area on our coordinate system to investigate which regions are most susceptible to denudation. The denudation region was selected because we hypothesized that areas near or around common locations of denudation were likely areas of characterized by cartilage loss. We explored the use of 3, 5, 7, 9 and 12 optimal locations. The 9 informative locations provided a good estimate of the overall cartilage change.

*Development of cartilage measurement software*

We developed cartilage measurement software to register knee MR image sets to these coordinate systems. In the first step the user indicates the most medial and lateral MR image slices within the knee. These images designate the minimum and maximum values of the vertical axis on the coordinate system. Next, the software automatically determines the MR image slices that contain informative locations, which is more responsive to cartilage denudation, based on the universal coordinate. On each of these slices the user then manually traces the bone-cartilage boundary (main paper Figure 2). The software then translates the curvilinear articular surface to a standardized horizontal axis and indicates the predefined informative locations so that the reader could measure the cartilage thickness at these points (main paper Figure 2). To co-locate the corresponding informative locations on baseline and follow-up images, we use dual screens to permit a visual comparison of the measures on baseline and follow-up MR images.

*Cartilage quantification at informative locations*

The CDI is defined as the sum of the thickness of the cartilage at each informative location multiplied by the cartilage length on that slice and the voxel size (mm3). One investigator (MZ) performed the measurement using the new approach among 83 knees.

*Statistical analyses*

Spearman’s correlations were calculated to test the association between baseline and longitudinal CDI score and alignment and bone marrow lesions (BML). The same associations were tested using traditional articular cartilage volume via manual segmentation compared with alignment and BMLs.

*Results*

The 83 knees included 47 (56.63% ) KL=2, 27 (32.53%) KL=3, and 9 (10.84%) KL=4 at baseline. As expected based on prior studies, we found that increased varus alignment appeared to be associated with lower cartilage volume in the medial tibio-femoral compartments though the p-value was 0.06, just missing the level of statistical significance. However, when evaluating the tibia or femur separately, this relationship was not observed. (Table 2)

When evaluating the relationship of cartilage volume with medial BMLs, we found that baseline medial tibial BMLs was strongly associated with baseline traditional cartilage volume in the medial tibia; however, we were not able to detect this relationship when evaluating the medial femur or the combined medial tibio-femoral compartment.

When using the CDI score, focused on 9 informative locations we found that the baseline and change of CDI score (medial tibia, femur, and tibiofemoral) were associated with baseline and longitudinal alignment and baseline BMLs (see table 2).

*Discussion*

Therefore, the CDI has better construct validity when compared to traditional cartilage volume assessments in the Vitamin D dataset. In the developmental dataset we found that 9 informative locations was the lowest number to approximate manually segmented cartilage volume change.

*Step 2: Transition to 3-Tesla MR Images*

*Dataset (n=82)*

To transition from 1.5-Tesla to 3.0-Tesla MRIs, we used a set of knees with baseline and 12-month follow-up 3D Dual Echo Steady State (DESS) images from the OAI. The 82 knees were selected from the OAI progression cohort, which included individuals with symptomatic knee OA in at least one knee. The baseline and 12-month follow-up 3D sagittal DESS images were manually segmented by iMorphics and the raw data, including the manual segmentation, as well as the processed cartilage volume assessments were made available through the OAI website: (<http://oai.epi-ucsf.org/datarelease/>).

*Procedure*

One investigator (MZ) performed the measurement using the approach developed in the initial step among 82 knees. We calculated Spearman’s correlations to test the association between CDI score and traditional cartilage volume using manual segmentation.

*Results*

The 82 knees included 2 (2.44% ) KL=1, 28 (34.15%) KL=2, 49 (59.76%) KL=3, and 3 (3.66%) KL=4 at baseline. Cross-sectional CDI scores were associated with traditional cartilage volume (r > 0.50; see table 3). Longitudinal change in tibial CDI score was correlated to tibial cartilage volume change (r = 0.36, p<0.01), but we did not observe a similar correlation in the femoral measurements.

*Discussion*

We hypothesized that the good cross-sectional associations between CDI score and traditional cartilage volume indicated that CDI was a reasonable reflection of the overall cartilage volume. However, weaker longitudinal findings may be attributed to the short follow-up period (12 months) that may have limited changes, especially for the femur (femur manual segmentation SRM=-0.0739, tibia manual segmentation SRM = -0.1382). Therefore, we developed a preliminary testing dataset in step 3, which included a different 98 knees with longer follow up, with 3D DESS MR images at baseline and 24-month follow-up.

*Step 3: Preliminary Testing*

*Dataset (n=98)*

To further evaluate the new method we selected 98 knees with baseline and 24-month follow-up 3D DESS MR images from OAI. We chose knees that had centrally-read semiquantitative radiographic readings as well as quantitative JSW measurements and HKA angles (noted in main paper). Among knees with complete data we selected knees with a diverse range of KL grades (Table 1) and HKA angles.

*Procedure*

One investigator (MZ) performed the measurement on baseline and 24-month follow-up MR images using the cartilage quantification method developed in the first step.

To explore the construct validity of the new cartilage quantification method we evaluated the cross-sectional and longitudinal associations between CDI score and HKA angle and joint space width (JSW). Specifically, we used Spearman rank correlation coefficients to evaluate the association between HKA angle and baseline CDI scores as well as CDI scores change. We also calculated Spearman rank correlation coefficients to assess the association between baseline medial JSW and baseline CDI scores, follow-up medial JSW and follow-up CDI scores as well as medial JSW change and change in CDI scores.

*Results and discussion*

The 82 knees included 16 (16.33%) KL=0, 15(15.31% ) KL=1, 16 (16.33%) KL=2, 41 (41.84%) KL=3, and 10(10.20%) KL=4 at baseline.

We found that the baseline CDI score (medial tibia, femur, and tibiofemoral) were associated with baseline JSW (Spearman r = 0.68 to 0.83, p <0.01) and HKA (Spearman r = 0.28 to 0.40, p <0.01 to p = 0.017), except for baseline tibial CDI score with HKA (Spearman r = 0.12, p = 0.25). Furthermore, changes in CDI score were associated with HKA (Spearman r = 0.26 to 0.41, p = 0.018 to <0.01) and changes in JSW (Spearman r = 0.30 to 0.38, p < 0.01).

During phase 3 we established segmentation rules that would promote good intra- and inter-tester reliability and evaluated the longitudinal performance of CDI score. Our results suggested that CDI score has good construct validity based on cross-sectional and longitudinal associations with static knee alignment and medial JSW. Therefore, we conducted a final phase to test intra- and inter-tester reliability and to confirm construct validity by exploring associations between CDI score and standard radiographic assessments.

*Step 4: Final Validation*

The detail of final validation step is in main paper.

**Table 1.** Descriptive characteristics for the samples from the 3 Steps

|  | Step 1 (n=83) | | Step 2 (n=82) | | Step 3 (n=98) | |
| --- | --- | --- | --- | --- | --- | --- |
| Age, mean (SD) | 63.54 (8.07) | | 60.6 (9.9) | | 60.8 (9.0) | |
| Women, n (%) | 50 (60.24) | | 41 (50) | | 53 (54.08) | |
|  | **Baseline**  **n (%)** | **Follow-up**  **n (%)** | **Baseline**  **n (%)** | **Follow-up**  **n (%)** | **Baseline**  **n (%)** | **Follow-up**  **n (%)** |
| Joint Space Narrowing Grade | | | | | | |
| 0 | No data | No data | 9 (10.98) | 9 (10.98) | 50 (51.02) | 43 (47.25) |
| 1 | No data | No data | 21 (25.61) | 18 (21.95) | 13 (13.27) | 11 (12.09) |
| 2 | No data | No data | 49 (59.76) | 46 (56.10) | 29 (29.59) | 23 (25.27) |
| 3 | No data | No data | 3 (3.66) | 9 (10.98) | 6 (6.12) | 14 (15.38) |
| Kellgren-Lawrence Grade | | | | | | |
| 0 | 0 (0.00) | No data | 0 (0.00) | 0 (0.00) | 16 (16.33) | 13 (14.29) |
| 1 | 0 (0.00) | No data | 2 (2.44) | 2 (2.44) | 15 (15.31) | 13 (14.29) |
| 2 | 47 (56.63) | No data | 28 (34.15) | 25 (30.49) | 16 (16.33) | 12 (13.19) |
| 3 | 27 (32.53) | No data | 49 (59.76) | 46 (56.10) | 41 (41.84) | 31 (34.07) |
| 4 | 9 (10.84) | No data | 3 (3.66) | 9 (10.98) | 10 (10.20) | 22 (24.18) |
| Note: n = number; SD = standard derivation | | | | | | |

**Table 2.** Spearman association between cartilage damage index, manual cartilage segmentation and alignment, BML (Step 1)

| Cartilage Measure | Alignment  (p-value) | | BML (Baseline)  (p-value) |
| --- | --- | --- | --- |
| Cross-sectional | | | |
| Femur CDI (Baseline) | | 0.30 (<0.01) | -0.34 (<0.01) |
| Femur cartilage volume (Baseline) | | -0.07 (0.04) | -0.07 (0.55) |
| Tibia CDI (Baseline) | | 0.26 (0.02) | -0.42 (<0.01) |
| Tibia cartilage volume (Baseline) | | -0.05 (0.64) | -0.28 ( 0.01) |
| Tibiofemoral CDI (Baseline) | | 0.31 (<0.01) | -0.50 (<0.01) |
| Tibiofemoral cartilage volume (Baseline) | | -0.21 (0.06) | -0.16 (0.16) |
| Longitudinal | | | |
| Femur CDI (Change) | | 0.26 (0.02) | No compare |
| Femur cartilage volume (Change) | | 0.27 (0.01) | No compare |
| Tibia CDI (Change) | | 0.23 (0.03) | No compare |
| Tibia cartilage volume (Change) | | 0.28 (0.01) | No compare |
| Tibiofemoral CDI (Change) | | 0.29 (<0.01) | No compare |
| Tibiofemoral cartilage volume (Change) | | 0.31 (<0.01) | No compare |
| Note: change = follow-up minus baseline; BML = bone marrow lesions  Lower CDI = greater damage, lower cartilage volume = greater damage, higher alignment measure = more valgus? | | | |

**Table 3. Association between cartilage damage index and manual cartilage volume (Step 2)**

|  | **Spearman association with manual volume (p-value)** |
| --- | --- |
| **Cross-sectional** | |
| **Femur CDI (Baseline)** | 0.60 (<0.01) |
| **Tibia CDI (Baseline)** | 0.63 (<0.01) |
| **Tibiofemoral CDI (Baseline)** | 0.65 (<0.01) |
| **Longitudinal** | |
| **Femur CDI (Change)** | -0.02 (0.88) |
| **Tibia CDI (Change)** | 0.36 (<0.01) |
| **Tibiofemoral CDI (change)** | 0.10 (0.41) |
| Notes: change = follow-up minus baseline; | |

**Table 4**. **Spearman association between CDI score and HKA, JSW (Step 3)**

|  | HKA  (p-value) | JSW (Baseline)  (p-value) | JSW (Change)  (p-value) |
| --- | --- | --- | --- |
| Cross-sectional | | | |
| Femur CDI (Baseline) | 0.32 (<0.01) | 0.68 (<0.01) | No compare |
| Tibia CDI (Baseline) | 0.12 (0.245) | 0.71 (<0.01) | No compare |
| Tibiofemoral CDI (Baseline) | 0.24 (0.016) | 0.71 (<0.01) | No compare |
| Longitudinal | | | |
| Femur CDI (Change) | 0.26 (0.018) | No compare | 0.30 (<0.01) |
| Tibia CDI (Change) | 0.41 (<0.01) | No compare | 0.38 (<0.01) |
| Tibiofemoral CDI (Change) | 0.35 (<0.01) | No compare | 0.36 (<0.01) |
